# Low-Waste Technology for High-Precision Connecting Rod Forging Manufacturing

**DOI:** 10.3390/ma18020443

**Published:** 2025-01-18

**Authors:** Łukasz Dudkiewicz, Marek Hawryluk

**Affiliations:** 1 Department of Metal Forming, Welding and Metrology, Wroclaw University of Science and Technology, Lukasiewicza Street 5, 50-370 Wroclaw, Poland; lukasz.dudkiewicz@pwr.edu.pl; 2 Schraner Polska, Lotnicza 21g, 99-100 Łeczyca, Poland

**Keywords:** hammer precision forging, FEM modelling, dynamic movements of die, high speed camera measurements, technology improvement

## Abstract

This study refers to the application of an advanced tool in the form of numerical modelling in order to develop a low-waste hot die forging technology to produce a connecting rod forging. The technology aims at ensuring a limited amount of the charge material is necessary to produce one forging, as well as minimizing forging forces, and thus the electric energy consumption. The study includes a verification of the current production technology, which constituted the basis for the construction and development of a numerical model. A new construction of the forging tools was developed, with an additional pre-roughing pass (0X). The new process consists of die forging in the pre-roughing pass (0X), the roughing pass (1X) and the finishing impression (2X). Numerical modelling was subsequently conducted with the use of the Forge 3.0 NxT software. A detailed analysis was conducted on the accuracy of the tool impression filling (including the pre-roughing pass) by the deformed material, the distribution of temperatures for the forgings and the plastic deformations, as well as the courses of forging forces and energy. The results were verified under industrial conditions and compared with the forgings obtained in the previous technology (a roughing pass and a finishing impression). As a result of introducing the pre-roughing pass 0X, the forces were distributed between three impressions, including the especially developed pre-roughing pass. It was confirmed that the abovementioned changes in terms of forging tool construction had a positive effect on relieving the roughing pass and the finishing impression as well as limiting the charge material, and they also lowered the process energy consumption by 10%. This study also validated the relevance of using FE modelling to verify processes under virtual conditions before being implemented under industrial conditions. Therefore, the proposed approach based on multi-variant numerical simulations can be successfully used to improve other manufacturing processes in terms of reducing energy and material consumption and increasing tool service life.

## 1. Introduction

Currently, in the face of high energy and utility costs, as well as the difficult geopolitical situation and the resulting risk of supply chain disruptions for various raw materials, there is a continuous pursuit of new manufacturing technologies for components and subassemblies, including connecting rods. These components serve as critical links in key machine elements [1]. Connecting rods are important and responsible elements of combustion engines, including those in cars, motorcycles and other machines and devices [2]. They have to be characterized by high mechanical properties, including tensile strength, yield strength and elongation. Such forgings also constitute an important safety element; thus, they have to demonstrate dimension and shape precision as well as lack of surface decarburization and a proper fiber arrangement [3], and should also be in line with the standards [4]. Their purpose is to convert the linear displacement of the piston into the rotational movement of the crankshaft [5]. For this reason, the connecting rod is viewed as the key component of the engine, which has a significant effect on its durability and efficiency. For these reasons, it is produced in a hot die forging process on hammers [6].

The technology of producing die forgings, which are connecting elements (such as connecting rods), still constitutes an unsolved problem as well as a big challenge. This is because, in the process of forging on hammers, an important role is played not only by the selection of the proper technological process parameters but also the proper placement and forming of the forged material in the subsequent die cavities [7]. To a large extent, this depends on the experience and skill of the operator but also on the difficult conditions present in the process, such as high temperatures, vibration, dusting, etc. The standard process for producing connecting rods consists of cutting the charge material to the appropriate length, followed by heating the material to the forging temperature. The next step is the actual die forging process, hot trimming and shot blasting. The connecting rods are then copper-plated, mechanically machined and undergo heat and chemical treatment [8]. Depending on the specific requirements of the clients, sometimes, cold calibration or an additional thermal treatment, i.e., softening or normalizing annealing, are also applied [9]. In order to increase the process efficiency and at the same time lower the unit cost, the forgings are produced in multiple systems on hammers. The cycle time of such a process is about 10 s. In the standard hot die forging process of producing connecting rod forgings, the flash constitutes 20–70% of the charge material. In order to lower the unit cost of the product in the form of a forging, the forging process should be realized in a closed die, so that near-net or net shape parts can be obtained [10,11]. In the case of forging without a flash, the distribution of the forging cavity volume must be precisely controlled to avoid overloading of the tool’s impression and to properly fill the forging cavity [12]. Although the technology of producing connecting rods through the process of hot die forging is well-known [13], the development of a manufacturing technology for serial and mass production, while maintaining appropriate shape and dimensional accuracy [14], is a challenging issue [15]. At the same time, it requires minimizing the amount of the charge material by limiting it in order to achieve the desired cost level of the product, i.e., the forging [16]. In the analyzed literature and scientific papers, there are a few research studies related to designing, analyzing and producing connecting rods through the process of hot die forging. In the article [17], the authors analyzed defects appearing in a large, forged connecting rod. First, a comprehensive analysis of the forming process of the forged S20S connecting rod was performed. Then, through tests and microstructural studies, the results obtained showed that the main defects were caused by sulphide and oxide inclusions. In the article [18], powder metallurgy was used to develop a new technology for manufacturing a connecting rod element. The tests carried out showed that the physical and mechanical properties of the connecting rod manufactured using this technology are better than those manufactured using the traditional method. In the article [19], the authors presented the results of experimental studies of the flash-free connecting rod forging process in a three-slider forging press, for which they developed an ideal preform in the wedge-cross rolling process. In order to develop a new technology for manufacturing connecting rods, in the work [9], numerical simulations were used in the design of the connecting rod forging process using the PT Creo 11 software (version 11, 2024), as well as the optimization of the initial die form and its analysis using numerical modeling. Interesting results concerning the development of the connecting rod manufacturing technology in China can be found in the work [9], where, based on experience in the development of specialist forging lines in recent years, progress in precision forging of connecting rods was presented. The presented approach included, among others, automatic feeding of billets together with a temperature control system, the use of 3D-CAD (SolidWorks 2024)/CAM (Mastercam 2024) programs for cross-rolling and reduction rolling of blanks and modern forging equipment [20,21]. The analysis carried out indicates that most of the research works in this area, for the development of innovative technologies for manufacturing or improving the forging processes of such elements, which are used for responsible machine parts, are supported by different programs and numerical modeling, which give the best results [22,23,24]. This is especially for parts manufactured by forging and dedicated to aeroengine turbines’ application [25,26], where these processes are supported by advance rolling [27,28]. This approach is also used to develop new forging technology [29] for the other materials like steel, for example, for chrome bronze [30], or even for composites on base Al alloys [31], as well as for forging quality measurements [32,33]. These advanced and combined methods are also eagerly used for improvements in the forging processes of connection rods [6,34,35]. In particular, the use of numerical modeling is currently an indispensable and frequently used tool for realizing fast, accurate and relatively reliable virtual experiments [36,37,38]. However, none of them refer to the production of a connecting rod forging with preliminary deformation on a hydraulic hammer without the use of a rolling mill, which has been proposed as an innovation in this research paper.

The aim of this study is to develop a low-waste technology for precision die forging of a connecting rod on a hydraulic hammer, using numerical modelling with FEM, by reducing the volume (diameter) of the charge material. This should, in turn, allow for an increase in the durability of the forging tools. The obtained results have been verified under industrial conditions.

## 2. Materials and Methods

**Figure 1:**
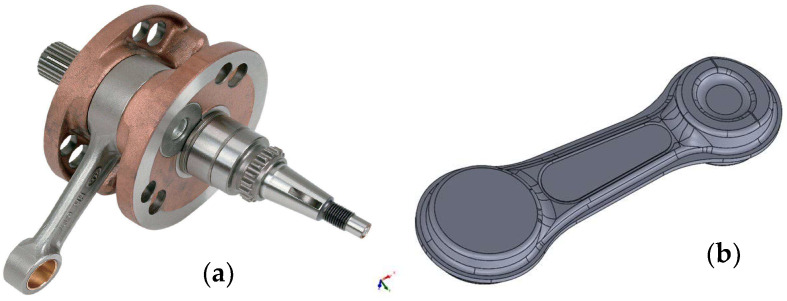
The view of (**a**) the solid of the complete crankshaft, and (**b**) a 3D drawing of the connecting rod.

The subject of the research was a forging with an elongated axis, i.e., a connecting rod, which is produced in a multiple, i.e., triple, system. Figure 1 below presents a view of the selected connecting rod.

Currently, the process of die forging is conducted with the use of a LASCO hammer with an impact energy of 20 kJ (LASCO Umformtechnik GmbH, Coburg, Germany). Precision forging is realized on open dies consisting of two impressions, i.e., the roughing pass and the finishing impression. The impact energy is 40% and 35.5% for the roughing pass and 26.5% for the finishing impression. The material used for the tools is hot work steel 1.2344. The material used for the forgings is steel 42CrMo4 with a diameter of Ø16 mm. The material is heated to the forging temperature in an electric furnace.

**Figure 2:**
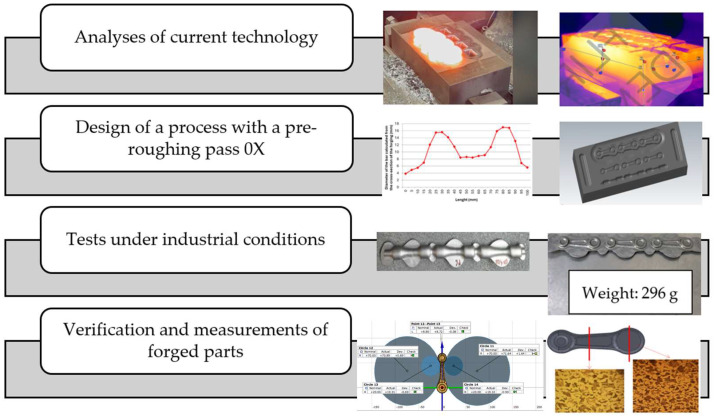
Flowchart of the methodology.

The complex research studies included the following tests, which are also presented in the flowchart of the methodology (Figure 2):

1. Analyses of the presently realized technology of precision forging in a triple system.
2. Development and design of a process of precision forging in a triple system with a pre-roughing pass 0X, together with advanced and detailed numerical simulations.
3. Tests under industrial conditions in order to verify the conducted changes and forging quality tests.
4. Measurements of the displacement of the upper and lower forging tools with and without guiding locks with respect to each other during the die forging process and their effect on the product quality.

In order to achieve the assumed goal, the following methods, techniques and measurement/testing tools were used:

- A complex analysis of the forging process, including the use of a thermovision camera Flir 840 (FLIR Thermal Studio Starter, Teledyne FLIR LLC, 27700 SW Parkway Avenue, Wilsonville, OR 97070, USA);
- Based on the current production technology and the necessary documentation, 3D solids, models and drawings based on the current technical documentation with the use of the CAD SolidWorks 2024 (Dassault Systèmes Sp.z.o.o, Krakov, Poland) and CAM Mastercam 2024 software (Zalco Sp. z o.o., Warsaw, Poland) were included;
- A numerical model was developed on the basis of the above data and the subsequent simulations were conducted with the use of the computation package FORGE 3.0 NxT in order to determine the key parameters and physical quantities and identify the most important problems;
- Geometry measurements of the forgings and a hardness analysis of the obtained forgings were carried out with the use of fine control and measurement equipment, including calipers, a micrometer, a Werth measuring projector, a Mahr XC20 contour gauge (MarSurf XC 20 software, Mahr Poland Sp. z o.o., Warsaw, Poland) and a Mitutoyo Strato 776 automatic measuring machine (MCOSMOS 5 software, Mitutoyo Poland Sp. o.o., Wroclaw, Poland). The hardness measurements were performed with the use of an EMCOTEST hardness tester (EMCO-TEST Prüfmaschinen GmbH, Kuchl, Austria).

## 3. Results and Discussion

### 3.1. Analysis of the Currently Realized Technology of Precision Forging in a Triple System

Connecting rods with an elongated axis are most commonly produced from a solid material with a large allowance, approximately 20–70%, especially in small-batch production, or with the use of rolling mills in serial and mass production. Rolling mills provide the possibility to form a preliminary preform, thus limiting the amount of the charge material. The current technology is implemented in a triple system. The material is formed in three strikes: two on the roughing pass and one on the finishing impression. The material is formed from a round bar of Ø16 mm. However, in the case of serial or small-batch production, the purchase of a rolling mill may not be economically justified. In such cases, the preliminary forming can be carried out on the given forging unit.

**Figure 3:**
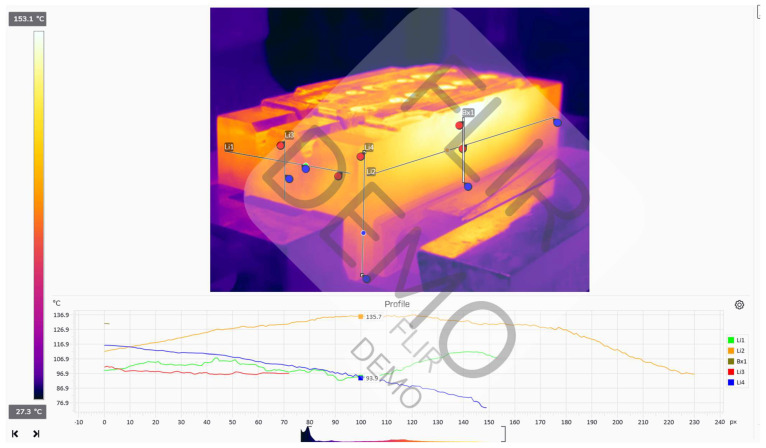
Analysis of the temperature distribution in the FLIR Thermal Studio application for the lower tool after the end of the heating process.

**Figure 4:**
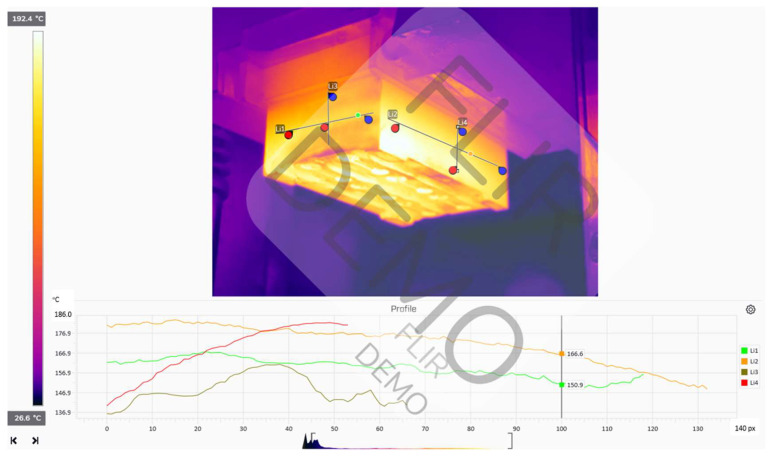
Analysis of the temperature distribution in the FLIR Thermal Studio application for the upper tool after the end of the heating process.

It was decided to record the temperature distribution after the heating process was completed and the heating device exited the position for both the upper and lower tools. The recorded temperature distributions have been presented in Figure 3 and Figure 4. 

For the manual process, a working temperature of the forging tools of 130–200 °C was recorded before the beginning of the forging process and after the end of the heating process. In turn, during the process, the working temperature equaled 150–220 °C. The thermograms show that the temperatures of both the lower and upper die change throughout the process. This, in consequence, hinders the realization of the process and the ensuring of its repeatability. Before the forging process begins, immediately after heating the forging tools, the upper die has a higher temperature than the lower one. This changes during the forging process, when the temperature of the upper forging tool decreases, while the temperature of the lower forging tool increases, due to its longer contact with the hot charge material in the form of a cylinder.

**Figure 5:**
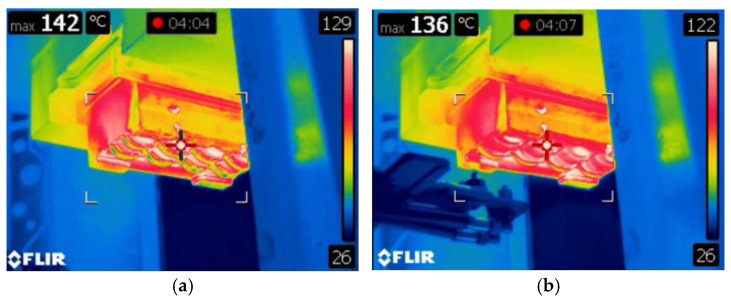
Measurement results from the thermovision camera for the process (**a**) before lubrication, and (**b**) after lubrication.

At the first stage of the conducted studies, automatic lubricant application system tests were also conducted. The performed tests made it possible to obtain the results of the thermovision measurements for the process. The data recording was carried out before the application of the lubricant and immediately after this process. Selected representative measurement results from the thermovision camera for the process have been presented in Figure 5.

It should be emphasized that the small temperature differences before the lubrication process are a noticeable phenomenon throughout the analyzed processes. We can notice that, as a result of the application of the lubricant, the tool temperature decreases by 6 °C. This confirms that a cyclic application of the lubricant affects the changeable working conditions and the tribological conditions.

The charge material had a temperature of 1250–1300 °C. It was heated in an electric furnace. Next, the operator collected the charge material and placed it onto the roughing pass, where two hammer strikes took place. The material was then transferred onto the finishing impression, where the third strike occurred. In one complete forging cycle, the tools are in contact with the charge material for a maximum of 0.5 s and during the remaining time, they cool down as a result of radiation and convection to the environment.

**Figure 6:**
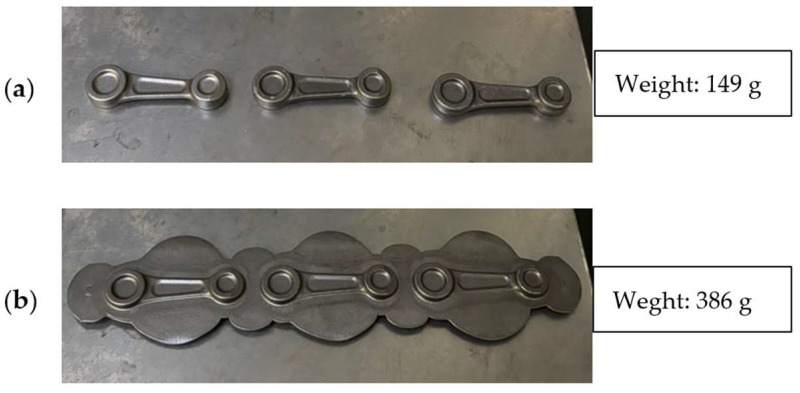
Views of a connecting rod forging: (**a**) a forging after trimming the flash, and (**b**) a forging with a flash.

It should be noted that the current production process, i.e., hot die forging, causes the formation of a large amount of waste material, i.e., the flash, which constitutes about 61% of the charge material, i.e., 237 g (Figure 6) and negatively affects the durability of the forging tools. The process is carried out by operators, blacksmiths, who have a significant influence on the result of the process. This is due to their individual predispositions, movements and rhythm. Therefore, as part of the optimized technology, changes will be introduced to increase the durability of the forging tools and reduce the charge material, ultimately decreasing the amount of waste. This will make it possible to reduce the charge material diameter from Ø16 mm to Ø14 mm and lower the energy and force parameters.

### 3.2. Development of Forging Tools with a Pre-Roughing Pass

The main aspect during the design of a new, low-waste technology is the selection of the number of tool impressions and their design to ensure the optimal distribution of deformation in the particular operations. This study refers to a solution using a forging hammer for the pre-roughing phase of the forging, while simultaneously reducing the charge material’s diameter to Ø14 mm and adding locks on the dies to increase the rigidity and compare the elaborated solution with the current technology.

**Figure 7:**
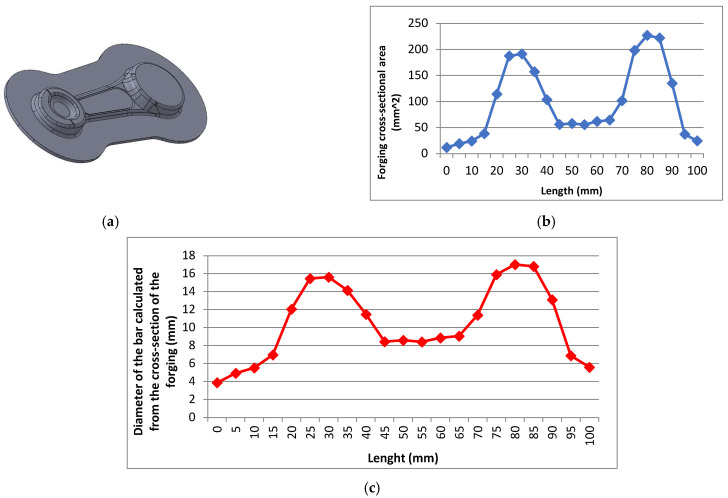
The view of (**a**) a forging model with the predicted flash, (**b**) a diagram of the forging’s cross-sections, and (**c**) the diameter of the rod calculated based on the cross-section of the forging.

The first step before the development of a pre-roughing pass was elaborating an ideal preform. To that end, calculations of the cross-section area of the connecting rod were made in the relevant areas. These calculations were conducted for one sector of the forging with a flash due to the multiplicity of the solution. The entire leaf consists of three identical products. The input data were assumed as a bridge with a width of 12 mm and a thickness of 1.5 mm, which is sufficient to freely fill the die during forging. Based on this, the cross-sectional areas for one of the three sections of the leaf forging were determined (it consists of three identical forgings with a flash) (Figure 7).

**Figure 8:**
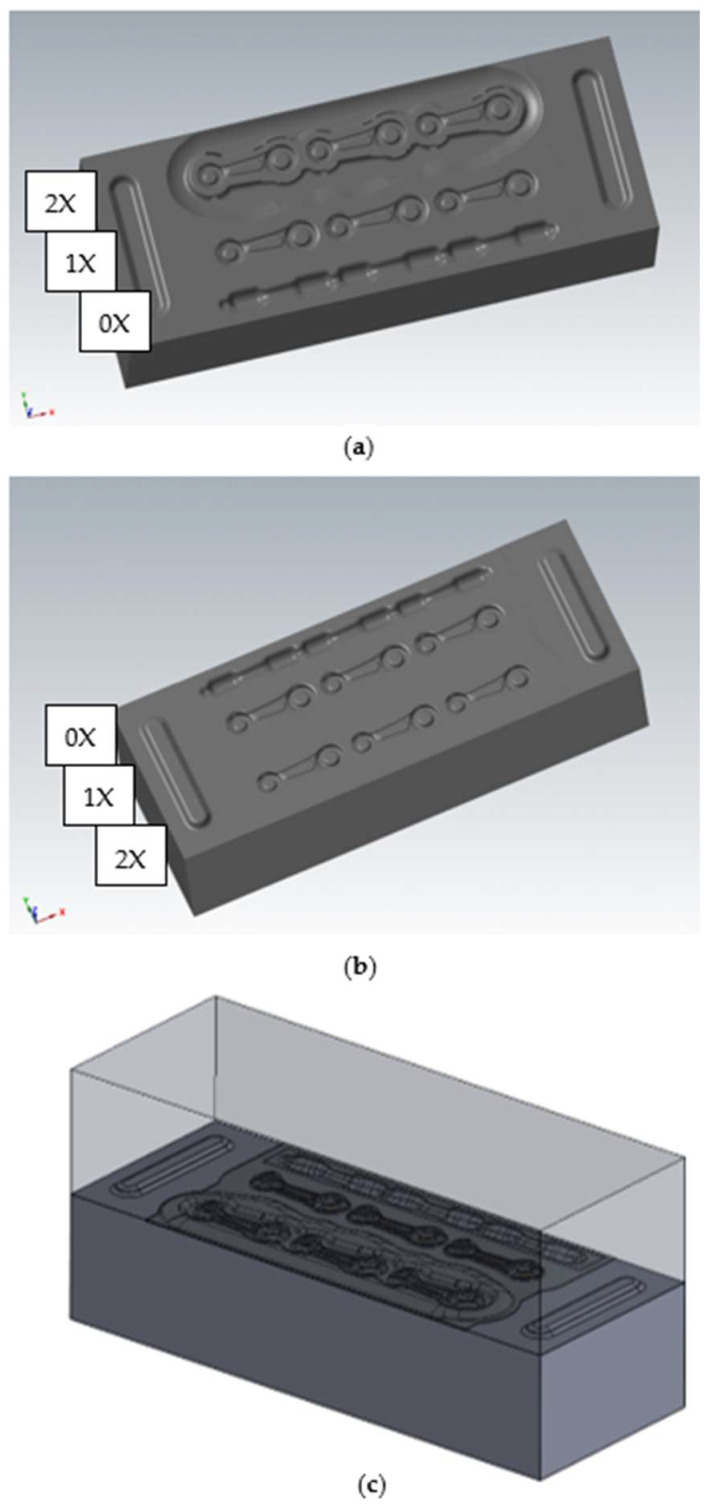
A 3D model of the forging tools with locks and a pre-roughing pass: (**a**) upper forging tool, (**b**) lower forging tool, and (**c**) a set of tools: upper and lower.

On the basis of the ideal preform, the pre-roughing pass was developed. The impression was designed for the diameter of the charge material in the form of a round bar of Ø14 mm (Figure 8, 0X). It was assumed that it would be a closed rolling impression, i.e., elliptical in shape. Based on this, the final shape of the pre-roughing pass with a bridge thickness of 1.5 mm was developed, similar to the shape of the roughing pass and the finishing impression. It should be emphasized that multi-variant numerical simulations with the use of FEM were conducted for bridge thicknesses of 1.2 mm, 1.5 mm and 2 mm. For a bridge thickness of 1.2 mm, very high forces were obtained, while for a thickness of 2 mm, the working impressions were not completely filled. For these reasons, it was concluded that a 1.5 mm bridge thickness is optimal for this type of forging tool geometry and the development of the technology.

**Figure 9:**
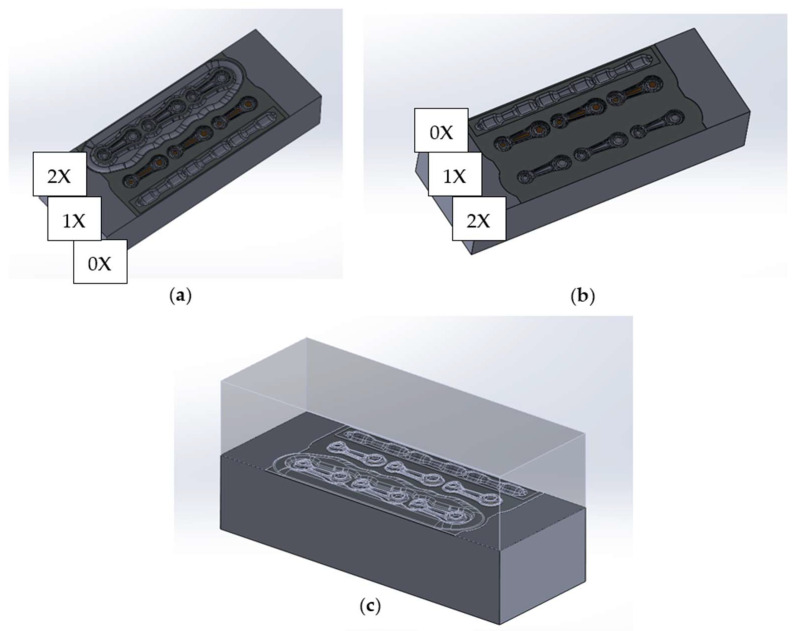
A 3D model of the forging tools without locks and with a pre-roughing pass: (**a**) upper forging tool, (**b**) lower forging tool, and (**c**) a set of tools: upper and lower.

The forging tools, together with the pre-roughing pass and the guiding locks, have been presented in Figure 8, while Figure 9 shows the forging tools without locks, for comparison with the current technology. As part of the ongoing work, the influence of the movement of the upper tool with respect to the lower tool during the forging process was verified, both with and without the locks.

### 3.3. Numerical Modelling of Hot Die Forging with a Pre-Roughing Pass

The forging process of the subject connecting rod in a multiple, triple system using a pre-roughing impression formed in one strike aims to reduce the forging forces and the amount of the used material. The reduction of the material diameter from Ø16 mm, which is not a standard round bar, to Ø14 mm, which is a standard product produced by steel mills, is a key change. During numerical modelling, particular attention was paid to the implementation of the pre-roughing and roughing pass to ensure proper filling of the working die cavities. Numerical modelling was carried out for both the variant of the forging tools with guiding locks and that without them.

**Figure 10:**
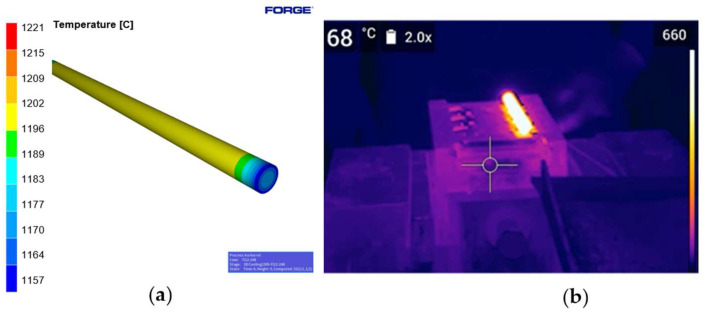
The view of (**a**) cooling of the charge material in the form of round bars after 6 s, and (**b**) the charge material in the form of a cylinder from the thermovision camera.

An assumption was made that the forging tools are non-deformable. The remaining assumed conditions were the same as those for forging in a manual mode. The charge material were bars made of 42CrMo4 [39,40] with a diameter of Ø14 mm. It was assumed that there would be one strike on the pre-roughing pass (0X), one strike on the roughing pass (1X) and one strike on the finishing impression (2X). The impact energy for 0X was 2.8 kJ, for the roughing pass 1X it was 4.8 kJ and for the finishing impression 2X it was 4.6 kJ. The forging temperature was 1300 °C and the cycle time was 13.5 s. It was divided into the subsequent steps: cooling for 6 s, forging on 0X—2.5 s, forging on 1X—2.5 s and forging on 2X—2.5 s. The total cycle time was 6 s + 2.5 s + 2.5 s + 2.5 s = 13.5 s. The forging process was realized with the use of an electric heater and a hydraulic hammer with a power of 20 kJ. The assumed tool temperature was 120 °C. The lubricant was water with graphite. The heat exchange was an average of 10 kW/(m^2^·K). The simulation results for the Ø14 mm bar diameter have been presented below. In Figure 10a, the cooling of the Ø14 mm bar is shown after 6 s, right before the pre-roughing process begins. Figure 10b presents a thermal image of the charge material. The figures clearly show that the temperature at the ends of the bar is lower than at the center, confirming the thermal instability along the entire length of the charge material in the form of a round bar.

**Figure 11:**
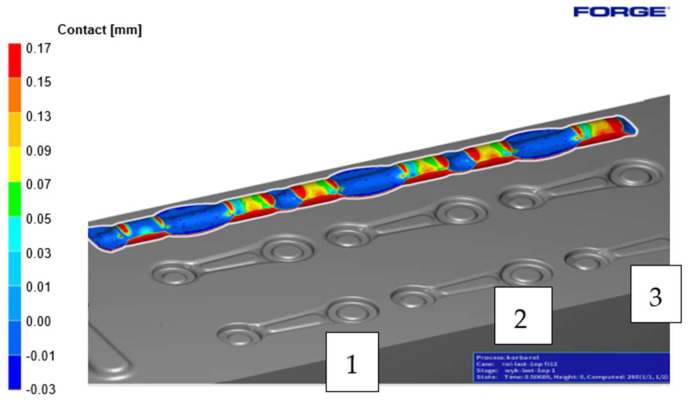
Contact of the pre-roughing pass 0X with the tools (number 1–3 mean cavity number).

Next, the charge material was placed on the pre-roughing pass 0X. Figure 11 shows the contact with the forging tool after the dies were completely closed. The bridge between the upper and lower forging tools is 1.5 mm (the opening). It is noticeable that the contact between the charge material and the tool is significantly better on the left seat, primarily in the area corresponding to the small eye of the connecting rod, compared to seats 2–3.

**Figure 12:**
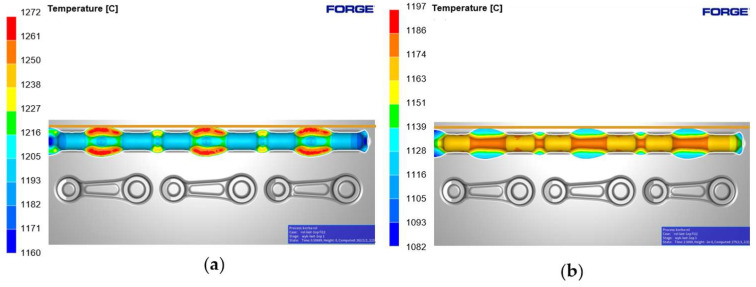
Temperature of the pre-roughing pass with the tools: (**a**) at tool closure, and (**b**) 2 s after forging.

Figure 12 shows the temperature of the semi-finished product at the moment of tool closure in Figure 12a and 2 s after the forging process in Figure 12b. A slight decrease in temperature can be observed. 

**Figure 13:**
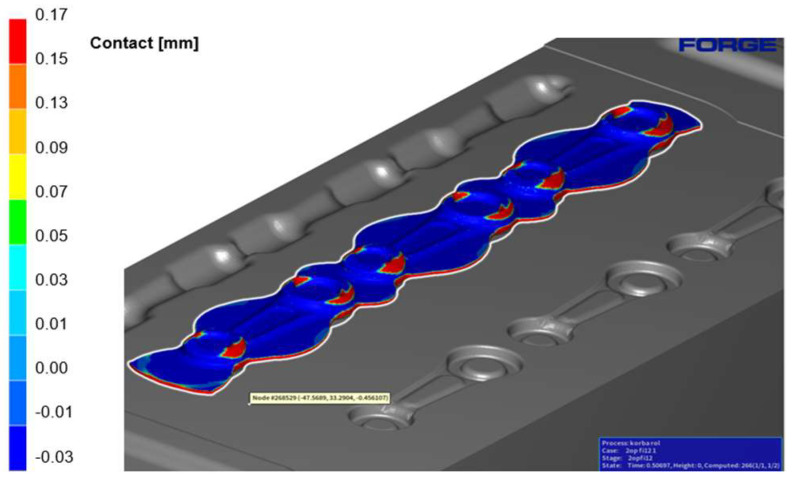
Full contact with the tools in the roughing operation 1X.

In Figure 13, the contact between the tool and the previously formed semi-product during the pre-roughing operation is shown. When the forging tools are fully closed with a bridge thickness of 1.5 mm, insufficient filling is visible in the radii of the large and small eye of the connecting rod. This area could undergo further modification and improvement to obtain better filling.

**Figure 14:**
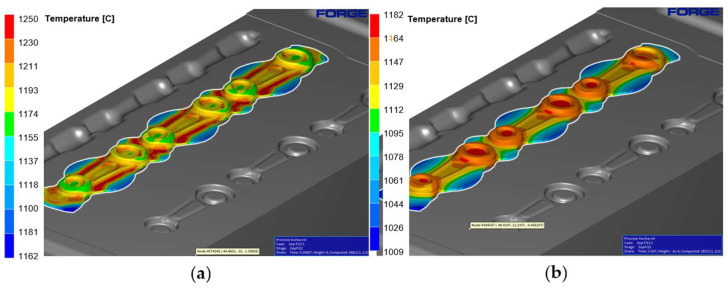
Temperature of the roughing pass 1X with the tools: (**a**) at tool closure, and (**b**) 2 s after forging.

The temperature distribution has been presented in Figure 14, both at full tool closure (Figure 14a) and 2 s after the forging process (Figure 14b). During the process, the temperature increases as a result of the conversion of plastic deformation work into heat, and in the pre-die forging operation, it reaches approximately 1065–1250 °C.

**Figure 15:**
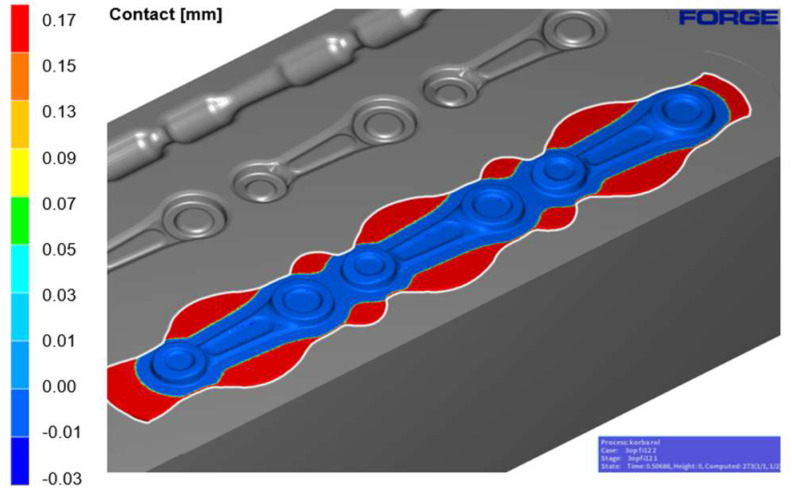
Contact of the finishing impression 2X with the tools, at tool closure.

Next, the semi-product from the roughing pass was formed in the finishing impression, corresponding to the final product—the forging, taking shrinkage into account. Figure 15 shows the contact at full closure of the tools. It can be seen that the finishing impression has been fully filled (blue color).

**Figure 16:**
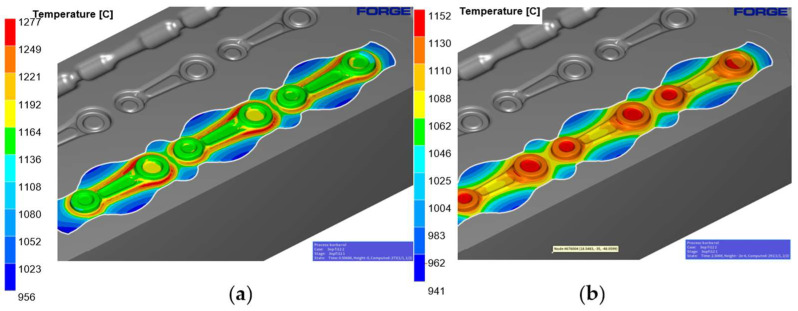
Temperature of the finishing with the tools: (**a**) at tool closure, and (**b**) 2 s after forging.

The temperature distribution for forming the semi-product during the finishing operation was the next step in the research. Figure 16a shows the temperature at the moment of full closure of the tool, while Figure 16b shows it 2 s after the forging process. The temperature at the end of the forging process after the finishing impression is about 955–1277 °C. In the models in question, a significant difference in the temperature distribution can be 

**Figure 17:**
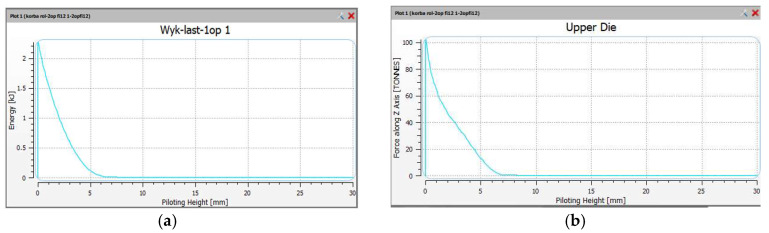
The views of the (**a**) impact energy—pre-roughing operation 0X, and (**b**) forging force—pre-roughing operation 0X.

Presented below is the forming of the forging forces and energy for the three impressions: the pre-roughing pass 0X, the roughing pass 1X and the finishing impression 2X. The approximate forming forces and forging energy are shown in Figure 17 for the pre-roughing operation. The energy was 2.8 kJ and the maximum forging force was 100 tons.

**Figure 18:**
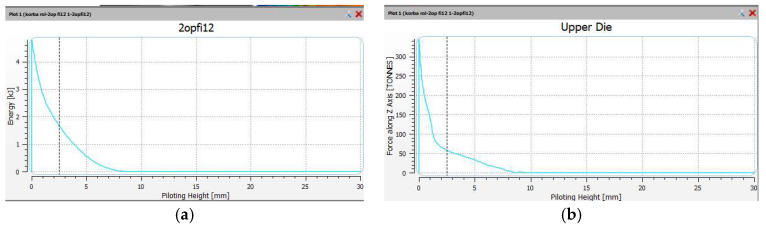
The views of the (**a**) impact energy, and (**b**) forging force, in the roughing operation 1X.

The forces and forming energy for the roughing operation 1X were also compared. The results are shown in Figure 18. In the roughing operation, there is one hammer strike. The energy was 4.8 kJ and the maximum forging force was 350 tons.

**Figure 19:**
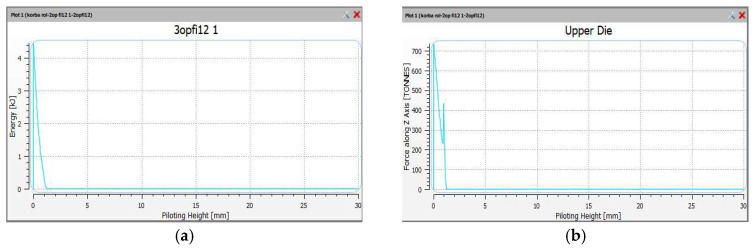
The views of the (**a**) impact energy, and (**b**) forging forces, in the finishing operation 2X.

Finally, the forces and forming energy for the finishing operation 2X were also compared. The results are shown in Figure 19. In the finishing operation, there is one hammer strike. The energy was 4.6 kJ and the maximum forging force was 720 tons.

**Figure 20:**
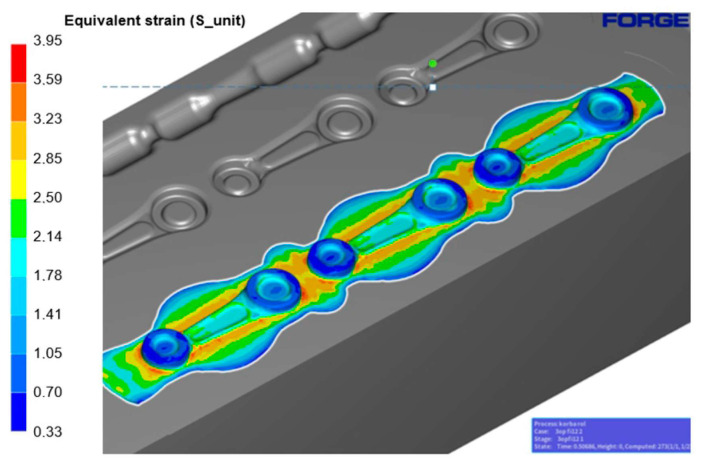
Results of the deformation simulations for the finishing operation 2X.

The forming force was reduced as a result of its distribution over three working impressions, compared to the standard technology, where two working impressions are used. The forming force was 100 tons for the pre-roughing pass, 300 tons for the roughing pass and 720 tons for the finishing impression. The energy needed to deform the detail in the finishing operation was 2.8 kJ for the pre-roughing pass, 4.8 kJ for the roughing pass and 4.6 kJ for the finishing impression. The energy increased from 2.8 kJ to 4.8 kJ. The plastic deformations have been presented for the finishing operation 2X in Figure 20. 

The obtained results indicate that the largest plastic deformations are located on the outer sides of the connecting rod eyes and near the connector, with the highest values of 3.3 observed in the area of the flash groove. Both the distributions of plastic deformation and the values are assumed and correct, which further confirms the validity of the conditions assumed in the numerical modelling.

For the variant of forging tools without guiding locks, the results of numerical modelling were the same (e.g., force, pressure and temperature values) as for the variant with guiding locks, because the clearance in the locks causes a lack of contact between these surfaces when the dies close under ideal conditions. Therefore, the article presents numerical simulations for the variant with guiding locks only.

## 4. Tests Under Industrial Conditions, Verification of Numerical Modelling and Quality Tests of the Forgings

The forging tools verified by means of numerical modelling were made through subtractive manufacturing and then tested under real industrial conditions. The process was realized with the use of a hydraulic hammer with a strike energy of 20 kJ. The table below presents the differences in the strike energy obtained within the numerical modelling for a bridge thickness of 1.5 mm and those used under industrial conditions in order to obtain forgings which are in agreement with the technical specification, also with a bridge thickness of 1.5 mm.

**Figure 21:**
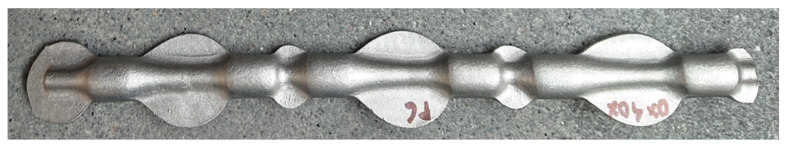
A semi-product made as a result of reforming of the charge material in the form of a bar on the pre-roughing pass 0X.

Based on Table 1 above, we can notice that the stroke energy from the numerical simulation is in agreement with the energy from the real industrial process, and it is about 6% higher. Figure 21 shows a reformed charge material in the form of a round bar on the pre-roughing pass 0X.

**Figure 22:**
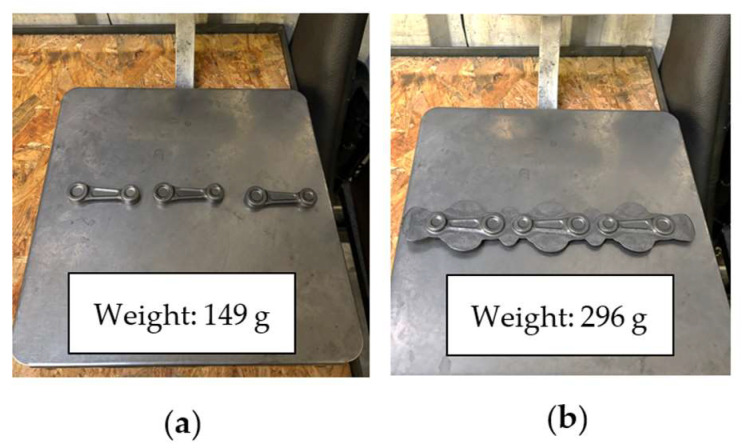
Views of the connecting rod forging in the form of a leaf: (**a**) a forging after flash trimming, and (**b**) a forging with the flash.

Figure 22 shows the results of measurements of the forging mass in the form of a leaf carried out with the use of the pre-roughing pass and the flash itself. As we can notice, the latter constitutes about 49% of the charge material. The pre-roughing pass was made from the diameter of the charge material, Ø14 mm. 

**Figure 23:**
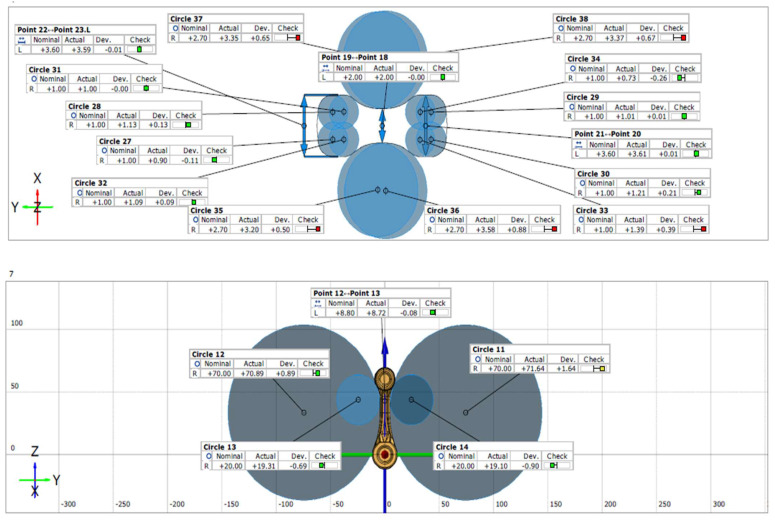
The scanning result of a connecting rod forging after the finishing operation 2X.

In order to verify the accuracy of the produced forgings, their measurements were carried out with special consideration of the radii of the large and small eyes of the connecting rod. In the first place, 3D scanning was performed on a test batch sampled from the forging process every 100 pieces. Figure 23 below presents an exemplary scanning result in the form of a color map of deviations for one leaf, randomly selected from the whole series. In order to conduct the measurements, one forging (leaf) was collected from the beginning of the process, and then three consecutive samples were collected every 100 items.

They were then cooled in air, trimmed of the flash and cleaned. A total of 12 trimmed forgings were obtained, which were then subjected to geometrical measurements. The obtained results are given in Table 2.

The above table confirms that the obtained measurement results are in agreement with the technical specification and are within the assumed dimensional tolerance scopes. This confirms that the process was elaborated correctly and can be successfully realized in this technology.

**Figure 24:**
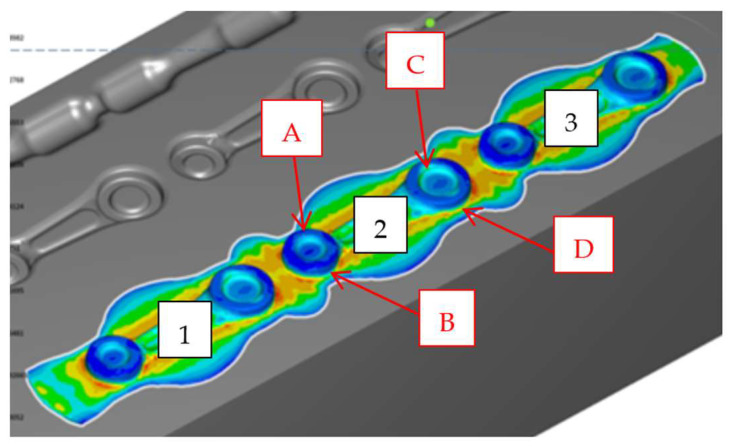
Designation of the measured radii R and forgings on the leaf (number 1–3 mean cavity number).

Next, the radii of the forgings were measured with the use of the Mahr XC20 contour graph. Figure 24 shows the information about the measured radii and their locations, while the measured values are presented in Table 3. The measurement results confirm that the radii are in accordance with the technical specification and that the working impressions are properly filled, including the critical areas.

Also, a verification was conducted of the hardness of the produced forgings, and the results were compared with those obtained with the previous technology, without a pre-roughing pass. The hardness measurement results are included in Table 4. The measurements were made by the Brinell HBW method, according to the guidelines included in the applicable standard [41]. The testing device was a hardness tester produced by EMCOTEST.

The average hardness for the forgings obtained as a result of die forging for the optimized technology is much higher than the average hardness using the Brinell scale for the current production technology, i.e., 9 HBW.

**Figure 25:**
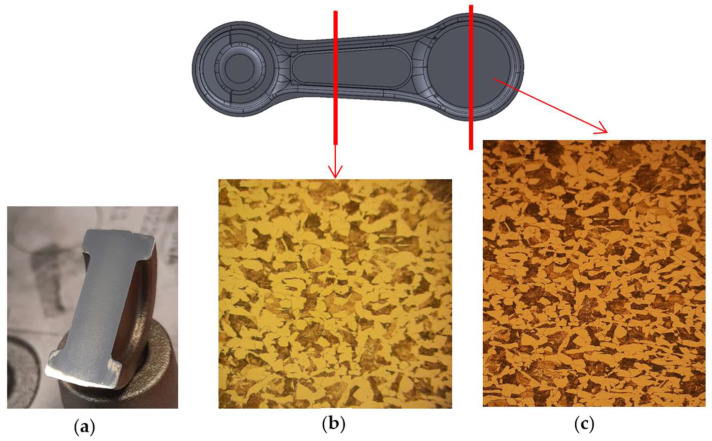
Results of microstructural tests for selected areas of the forging: (**a**) a view of the forging prepared for the tests, (**b**) the microstructure of the connecting rod link, and (**c**) the microstructure of the large eye of the connecting rod.

What is more, in order to verify the accuracy of the realized investigations and the elaborated technology, verification of the microstructure of the produced forging with a pre-roughing pass with respect to the forging obtained in the current technology was carried out. The microstructure was revealed by means of etching with a 5% nital reagent and the observation of the microstructures was performed on a laser microscope, Keyence VHX 6000, with magnifications of 100× and 200×. Figure 25 shows the microstructure for the connecting rod link and the large eye.

Figure 25 shows a typical ferritic–pearlitic microstructure for a forging in the newly-developed technology with a pre-roughing pass. For the standard technology, a ferritic–pearlitic structure was obtained as well, which confirms the agreement of the performed tests and the lack of an effect of the pre-roughing pass addition on the results of the forging process and the produced forging.

## 5. Measurements of the Displacements of the Upper and Lower Forging Tools With and Without Guiding Locks During the Die Forging Process and Their Effect on the Shift of the Forgings

**Figure 26:**
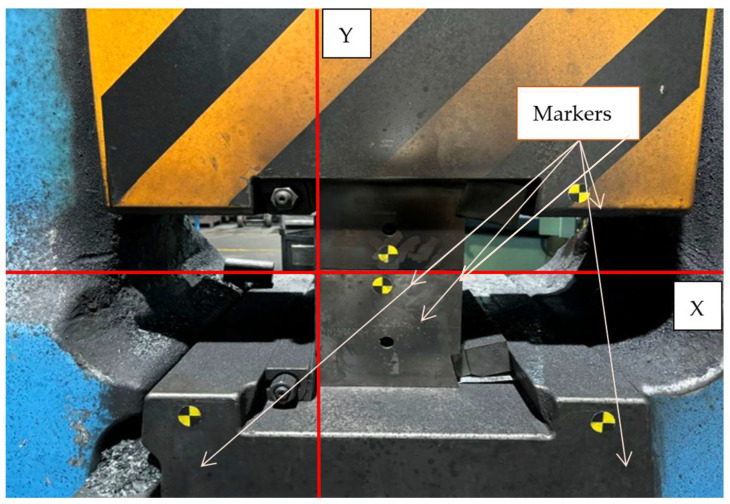
A view of the measurement system of the forging tools mounted on the adapters.

During the tests, measurements were performed of the displacements of the upper and lower forging tools in a construction with guiding locks (Figure 8) and without their use (Figure 9) in relation to each other. They aimed at verifying the clearances of the whole system, upper forging tool–lower forging tool, in reference to the displacement recorded on the forgings. Figure 26 shows a view of the processing area. Markers were mounted on the forging tools and the adapters as reference points. The measurements were conducted in the Y direction of the ram’s movement and in the X direction, i.e., the horizontal axis.

**Figure 27:**
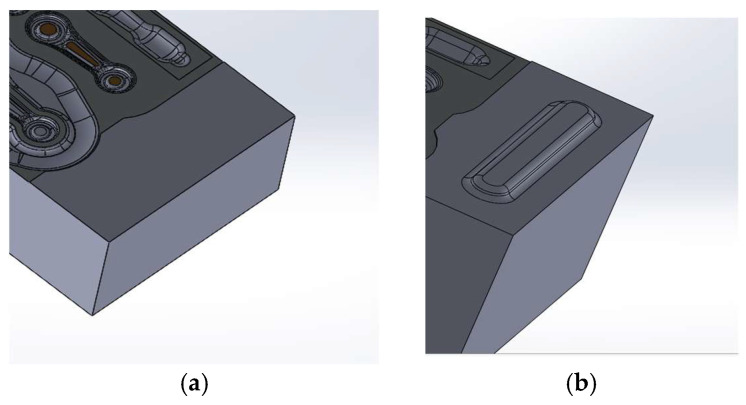
Views of (**a**) forging tools without locks, and (**b**) the lock of the lower forging tool.

Figure 27 presents a view of an upper and lower forging die in order to illustrate the shape of the locks. Their height equals 5 mm, their length is 90 mm and their width is 20 mm (in the widest area). The clearance on the locks equals, according to the developed geometry, 0.06 mm.

**Figure 28:**
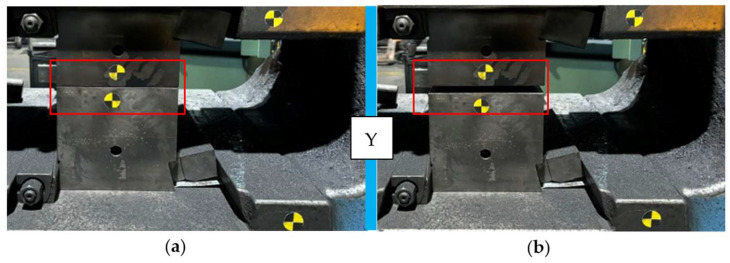
Selected frames at the subsequent forging stages pointing to the moment of opening of the dies: (**a**) contact of the upper forging tool with the lower one, Y = 0, and (**b**) 4 ms after the opening of the dies, Y = 10 mm.

As a result of these tests, selected frames at the subsequent forging stages are presented in Figure 28 below, pointing to the moment of the opening of the dies with locks.

**Figure 29:**
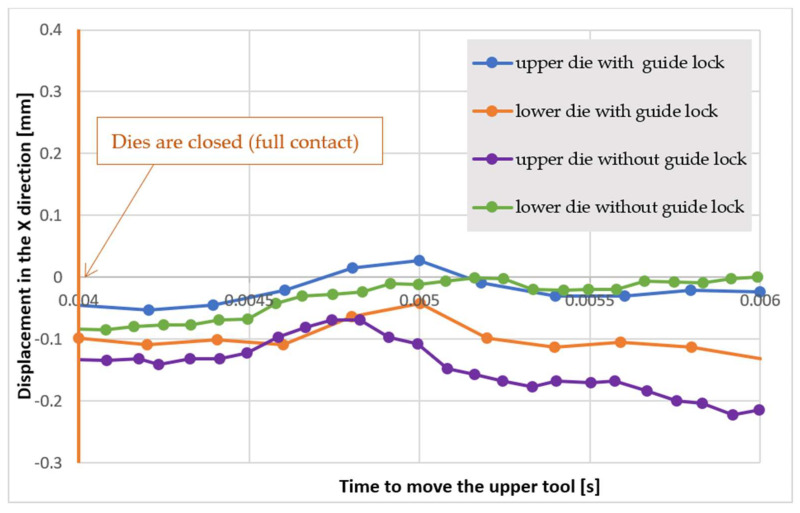
A diagram showing the relation between the displacement of the upper and lower forging tools and the adapters with respect to each other in time.

As a result of these tests, the displacement of the tools and the adapters with respect to each other was determined, as shown in Figure 29.

Figure 29 shows a diagram which presents the time after which the forging tools lose contact with each other on the locks. As we can notice, in the case of the forging tools with locks, the contact is lost after about 2 ms, that is, after the upper die is lifted with respect to the lower die by about 5 mm. In the case of the forging tools without locks, there is no additional guidance and the contact is lost right away. Also, the displacement of the dies with respect to each other equals a maximum of 0.107 mm in the whole scope of the ram’s movement, whereas that of the adapters is a maximum of 0.210 mm. The displacement of the forging tools with respect to each other is repeatable. The application of locks causes a reduction of the displacement of the forging tools with respect to each other, especially to their heights, with a reduction of 5 mm compared to the forging tools with locks. In the case of fast and dynamic processes, such as die forging on hammers, the use of guided locks is recommended to ensure the proper realization of the process.

Table 5 below shows the results of displacement for forging tools with guided locks and without their use, for five elements sampled every 50 items.

As we can see from the table above, the average displacement of the forgings for forging tools with guided locks is 0.08 mm, while for tools without guided locks, it is 0.128 mm. The guided locks used in the construction of the forging tools positively affect the displacement value of the forgings, thus improving their quality.

## 6. Comparison of the Newly-Developed Technology with the Standard Technology

Table 6 compares the newly-developed technology with the standard technology in order to analyze the economic benefits that can be gained from its use.

As we can see from the table above, the newly-developed technology brings many benefits, including reducing the diameter of the charge material, lowering the forging forces, and, as a result, providing economic advantages in the form of lower production costs. A lower diameter of the charge material results in reduced costs of material purchase, as well as lower costs for heating a smaller mass to the forging temperature, which helps reduce the electricity consumption. A lower mass of the charge material (Ø14 mm instead of Ø16 mm) makes it possible to apply lower forging forces, which lowers the consumption of electric energy by the hammer. What is more, reducing the input weight allows for a reduction of the pressures exerted by the forging tools, which increases their operational durability.

## 7. Summary and Final Conclusions

This study presents the results of the development of a new hot die forging process technology in a multiple system for a forging with an elongated axis, with a particular emphasis on reducing the amount of charge material required for its production. This is one of the proposed solutions for the forge to reduce the unit cost of the forging without the need to purchase additional, expensive equipment, such as a roller. The obtained results from this research have distinct applications as well as scientific import. The recipients of this acquired knowledge and outcome are primarily die forges as well as different research centers. A developed solution was proposed in the form of adding an additional pre-roughing pass in order to preliminarily shape the charge material into a round bar. This solution allowed for a reduction of unit pressures from two to three working impressions, as was previously used in the existing technology. As a result, this should, in the authors’ opinion, also positively affect the durability of the forging tools, extending their operational lifespan. For this purpose, numerical modelling was used with the Forge 3.0 NxT software package. The research consisted of a complex analysis of the production technology, as well as the proposal and design of a new forging tool construction. The verification was initially carried out in the virtual environment of the Forge 3.0 NxT software. Subsequently, the results were verified under industrial conditions with the use of a forging hammer with a pressure of 20 kJ. An agreement was achieved between the virtual test results and those obtained under industrial conditions. The forgings were verified for dimensional and shape accuracy, and it was confirmed that the change in technology did not negatively affect the quality of the forgings. The results confirmed the validity of the research and showed preliminary positive outcomes. The hardness of the forgings for the newly-developed solution is similar to that obtained with the previous manufacturing technology. Based on this, the most important final conclusions were drawn:

- The introduced technological improvements allowed for a reduction in the preform diameter from 16 mm to 14 mm, which consequently led to a decrease in mass, thus saving material and costs.
- The numerical simulations conducted for the variants of forging tools with and without guiding locks show no differences in the obtained results (e.g., force values, pressures and temperatures) because they simulate ideal conditions. In industrial processes, however, their positive effect on the process stability and forging displacement can be observed. The use of guiding locks in forging tools positively affects the realization of the technological process.
- The microstructural studies confirmed that the pre-roughing pass does not negatively affect the properties of the forging and confirmed that its structure is consistent with that of a forging without the pre-roughing pass. The expected and typical ferritic–pearlitic microstructure for forgings, in agreement with the technical specification, was obtained.
- The results obtained during the verification under industrial conditions for selected mechanical properties (hardness) and the dimensions of the geometric features are in agreement and correlate with the previous technology.
- The measurements of the displacement of the forging tools with respect to each other, together with the measurements, confirmed that, at the height of the locks (i.e., 5 mm), the displacement in the *X*-axis is stable and does not exceed 0.05 mm, despite the clearance in the locks resulting from the developed geometry, which is 0.06 mm. The use of guiding locks positively affects the displacement value and the quality of the produced forgings.

The results of the conducted numerical studies point to the significant potential of the use of numerical modelling methods and the agreement of the results obtained from FEM and under industrial conditions. These methods make it possible to reduce time and costly trials, which, in the current geopolitical situation of forging, is a crucial factor when selecting a supplier, particularly the time needed to produce the first trial batch. It is important to emphasize that the newly-developed technology brings numerous technical and technological benefits (low-waste technology) and, most importantly, provides concrete economic advantages in terms of the cost of producing die forgings.

## Figures and Tables

**Table 1:** Results of numerical simulations and the forging process realized under industrial conditions (a bridge thickness with closed dies of 1.5 mm).

Number of Hammer Strokes/Tool Impression	Energy from Numerical Simulations	Hammer Stroke Energy Under Industrial Conditions
0X	2.8 kJ	2.6 kJ
1X	4.8 kJ	4.5 kJ
2X	4.6 kJ	4.4 kJ

**Table 2:** The measurement results of selected representative characteristics.

Sample No.		Diameter Ø22 ± 0.1 (mm)	Diameter Ø19 ± 0.3 (mm)	Thickness 10.8 + 0.6 (mm)	Thickness 3.3 ± 0.2 (mm)	Distance from the Axis 53 ± 0.1 (mm)	Visual Inspection
1	1	21.98	19.03	11.09	3.29	53.038	OK
	2	21.94	19.02	11.11	3.31	53.008	OK
	3	21.95	19.00	11.09	3.28	52.969	OK
2	4	21.92	18.98	11.08	3.27	52.990	OK
	5	22.02	19.09	11.09	3.29	53.019	OK
	6	21.95	18.99	11.09	3.28	52.992	OK
3	7	21.95	18.99	11.10	3.29	52.970	OK
	8	21.97	19.02	11.09	3.29	53.000	OK
	9	21.94	18.98	11.11	3.30	52.975	OK
4	10	21.96	19.00	11.11	3.30	52.965	OK
	11	21.93	18.97	11.08	3.26	52.950	OK
	12	21.94	18.97	11.08	3.28	52.954	OK

**Table 3:** Measurement results of the radii for the small and large eyes of the connecting rod.

Sample No.		Small Eye Left Side R1 ± 0.5 (A) (mm)	Small Eye Right Side R1 ± 0.5 (B) (mm)	Big Eye Left Side R1 ± 0.5 (C) (mm)	Big Eye Right Side R1 ± 0.5 (D) (mm)
1	1	0.95	0.97	0.92	0.91
	2	1.03	1.05	0.98	0.95
	3	0.99	1.02	1.02	1.04
2	4	0.98	0.97	1.13	1.11
	5	1.05	1.02	1.02	0.98
	6	1.19	1.15	1.01	1.04
3	7	0.93	0.97	0.96	0.98
	8	0.97	0.98	0.93	0.95
	9	1.09	1.03	1.16	1.14
4	10	1.12	1.09	1.04	1.01
	11	0.94	0.96	0.97	0.99
	12	1.05	1.08	1.04	1.07

**Table 4:** Hardness measurement results of representative forgings after forging.

Sample No.	Average Durability of a Forging Using the Previous Technology (2 Impressions: 3 Strokes)	Average Durability of a Forging Using the New Technology (3 Impressions: 3 Strokes)
1	218 HBW	225 HBW
2	195 HBW	205 HBW
3	208 HBW	230 HBW
4	199 HBW	203 HBW
5	221 HBW	217 HBW
6	205 HBW	219 HBW
7	201 HBW	212 HBW
Average	207 HBW	216 HBW

**Table 5:** Measurements of the displacement of forgings in the version with guided locks and without their use.

Sample No.	Lateral Displacement in the *X*-Axis (mm)	
	Forging Tools with Guiding Locks	Forging Tools Without Guiding Locks
1	0.07	0.13
2	0.06	0.15
3	0.10	0.12
4	0.09	0.10
5	0.08	0.14
AVG	0.08	0.128

**Table 6:** Comparison of technologies.

Parameter	Standard Technology	Newly-Developed Technology	Improvement Percentage
Charge material diameter	16 mm	14 mm	−12.5%
Input weight	386 g	296 g	−23%
Material cost for producing one leaf (assuming a material price of 4 PLN/kg)	1.54 PLN/per leaf	1.18 PLN/per leaf	−23%
Weight of flash	237 g	147 g	−38%
Electric energy consumption *	40 kWh/h	36 kWh/h	−10%
Cycle time	10 s	10 s	0
Number of hammer strikes to produce one leaf	3	3	0
Strike forces 1	8 kJ	2.6 kJ	−67.5%
Strike forces 2	7.1 kJ	4.5 kJ	−37%
Strike forces 3	5.3 kJ	4.4 kJ	−17%
Durability of forging tools (without nitriding)	3000 items	3800 items	+27%

* The average amount of kWh consumed during 8 working hours. The measurement was taken with the use of an electricity meter for the work socket, i.e., the forging unit and the induction heater.

## Data Availability

The original contributions presented in this study are included in the article. Further inquiries can be directed to the corresponding author.
